# The Temporal Experience of Pleasure Scale (TEPS): Exploration and Confirmation of Factor Structure in a Healthy Chinese Sample

**DOI:** 10.1371/journal.pone.0035352

**Published:** 2012-04-18

**Authors:** Raymond C. K. Chan, Yan-fang Shi, Man-kin Lai, Yu-na Wang, Ya Wang, Ann M. Kring

**Affiliations:** 1 Neuropsychology and Applied Cognitive Neuroscience Laboratory, Key Laboratory of Mental Health, Institute of Psychology, Chinese Academy of Sciences, Beijing, China; 2 Graduate School, Chinese Academy of Sciences, Beijing, China; 3 Network for Health and Welfare Studies, The Hong Kong Polytechnic University, Hong Kong Special Administrative Region, China; 4 Department of Psychology, University of California, Berkeley, California, United States of America; University of California San Diego, United States of America

## Abstract

**Background:**

The Temporal Experience of Pleasure Scale (TEPS) is a measure specifically designed to capture the anticipatory and consummatory facets of pleasure. However, few studies have examined the structure of the measure in non-Western samples. The current study aimed to evaluate the factor structure and psychometric properties of the TEPS in a Chinese sample.

**Methods:**

We administered the Chinese version of the TEPS to 2275 healthy Chinese college students. They were randomly split into two sub-samples. The first sub-sample was used for exploratory factor analysis (EFA) to examine the structure of the TEPS in a Chinese sample. The second sub-sample was used as a validation sample for the identified structure from the EFA and confirmatory factor analysis (CFA) was adopted.

**Results:**

Results of the EFA suggested a four-factor model (consummatory contextual, consummatory abstract, anticipatory contextual, and anticipatory abstract factors) instead of the original two-factor model (consummatory and anticipatory factors) ascertained from Western samples in the United States. The CFA results confirmed these results in the second sub-sample. Internal consistency and test-retest stability of the TEPS factors were good.

**Conclusions:**

The TEPS has four factors among Chinese participants. Possible reasons for cultural difference and potential applications of the TEPS for cross-cultural comparison are discussed.

## Introduction

Anhedonia is one of the negative symptoms of schizophrenia and is also a key feature of depression associated with social functioning in these patients [1], [2]. Anhedonia refers to the loss or a reduction of the ability to experience pleasure [3]. In addition, similar emotional and personality disturbances have also been demonstrated in individuals at-risk for psychosis (c.f. [4]) and sub-clinical individuals with depressive symptoms (e.g., [5]). Klein [3] argues that anticipatory pleasure involves a feeling of wanting and is associated with motivation and goal-directed behavior, whereas consummatory pleasure involves a feeling of liking and is associated with satiation, or a resolution/fulfilling of a desire. Empirical evidence from social cognitive neuroscience and affective science also supports the distinction between consummatory pleasure and anticipatory pleasure [6]–[12].

However, most of the measures of anhedonia are limited to clinical rating scales and self-report trait questionnaires that adopt a unitary concept, particularly the in-the-moment experience of pleasure or consummatory pleasure (c.f. [4]). Anticipatory pleasure has not been as thoroughly studied. Despite the fact that many studies employ questionnaire based methods to assess trait positive emotion (e.g., [13]–[15]) and in-the-moment experience, very few scales or questionnaires have been designed to capture both anticipatory and consummatory constructs of pleasure.

Gard et al. [16] developed the Temporal Experience of Pleasure Scale (TEPS) to specifically capture these two distinct constructs of anticipatory and consummatory pleasure. It is a short questionnaire with 18 self-reported items (10 items for anticipatory pleasure, 8 for consummatory pleasure) allowing for easy administration. Examination of the scale factor structure in North American samples showed that the TEPS comprised the hypothesized 2-factor model for anticipatory and consummatory pleasure among 4 independent samples of university students [16]. Gard et al. [17] also demonstrated that patients with schizophrenia anticipated less pleasure from future activities than healthy controls during the course of daily life, and thereby, provided further evidence for the deficit of anticipatory pleasure in schizophrenia.

However, all the aforementioned findings were derived from Western samples. Given the potential for cultural differences of emotional experience between Western and Eastern people, the two factors in the TEPS may not fully capture cultural influences. For example, Chinese culture emphasizes the internal and external harmonious conditions between self and external environment as well as body and mind, i.e., the concept of *Yi-Yang* and *Taoism* [18]. Individuals are obligated to maintain their own internal harmonious condition through adjusting themselves to “context”. According to this belief, harmony is more or less equivalent to pleasure. Chinese people report lower frequency and intensity of emotional experience as compared to their Western counterparts [19]–[21]. One pilot study using the Chinese version of TEPS [22] showed impressive clinical utility of this scale in differentiating patients with schizophrenia characterized by negative symptoms from those patients without negative symptoms. However, the psychometric properties of the Chinese version of TEPS have not been fully examined.

The current study aimed to examine the factor structure of the TEPS in a large Chinese sample of healthy people. Given the cultural differences between the East and the West, we hypothesized that Chinese people would take less account of the difference in context between present and future time points when they prospect the future, as opposed to their Western counterparts. The secondary aim was to evaluate the internal consistency and test-retest reliability of the translated TEPS at a 4-week interval

## Methods

### Participants

This study involved three samples. Two thousand two hundred and seventy five undergraduates from four colleges/universities in Guangzhou and Beijing were split randomly into samples A and B. These colleges/universities were Guangdong Jidian Polytechnic, Sun Yat-Sen University at Guangzhou, Beijing Institute of Clothing Technology and Beijing Forestry University. Sample A consisted of 909 men and 247 women (mean age = 19.34 years, SD = 0.98), while sample B consisted of 870 men and 249 women (mean age = 19.36 years, SD = 0.98). There were no significant differences between the samples in gender, age and years of education. Both samples A and B were used to examine the factor structure of the TEPS. Participants in sample C were college students from Sun Yat-Sen University at Zhuhai and comprised 21 men and 35 women (mean age = 19.79 years, SD = 0.87). Individuals in sample C completed the TEPS on two occasions with a 4-week interval in between to assess test-retest reliability. All participants were native Mandarin speakers.

### The Chinese version of TEPS

The original English version of TEPS [16] is an 18-item, 6-point-Likert-format measure of anticipatory pleasure and consummatory pleasure. The current study followed the guidelines suggested by Beaton et al. [23] for cross-cultural translation of self-report measures. A panel comprised of 2 doctorate degree experts in psychology assessed these 18 items and found 2 of them (Item 5“I love it when people play with my hair” & item 11“When I'm on my way to an amusement park, I can hardly wait to ride the roller coasters”) to be a poor fit with Chinese culture traditions. The situation described in item 5 could be deemed offensive because most Chinese people consider the head to be an important body part referring to one's dignity and it's thus “to be sacred and inviolable”. For item 11, roller coasters might be popular among young people but not older people. For these considerations, we added two new items into the scale, one anticipatory and one consummatory (items 19, and 20; all items shown in Table 2). The final Chinese version of TEPS comprises 20 items with the same response format as the original English version, i.e., using a 6-point Likert scale (from 1 = *very false for me* to 6 = *very true for me*).

### Ethical Statement

The current study was approved by the ethics committee of the Institute of Psychology, Chinese Academy of Sciences in Beijing. Written consent was obtained from participants before the administration of the questionnaires.

### Data analysis

A 2-stage factor analysis approach was adopted to examine the factor structure of the translated TEPS. Sample A was analyzed using exploratory principal component factor analysis (EFA), whereas Sample B was analyzed using confirmatory factor analysis (CFA) in order to validate the identified factor structure from the EFA. Violation of normality in the variables is a problem with the maximum likelihood estimation method employed by CFA [24]. The Satorra-Bentler [25] and Bollen-Stine bootstrapping [26], [27] methods were adopted to overcome this problem in the current study. The Satorra-Bentler approach rescales chi-square statistics and estimates robust standard errors [25], whereas the Bollen-Stine bootstrapping method re-samples the data to calculate chi-square model fit statistics corrected for bias and estimates standard errors for significant tests [26], [27].

All the data preparation and EFA analyses were performed using SPSS 15.0 and the CFAs using the Satorra-Bentler approach were performed with LISREL 8.70 [28]. The Bollen-Stine bootstrap method was performed using the modified method for missing data in EQS 6.1 under the name Beran-Stine-Bentler bootstrapping procedure [29]. The number of bootstrap samples was selected to maximize accuracy of the evaluation of model fit [30], [31]. A moderate to large bootstrap sample size (N = 200 or above) was suggested [31], and a sample of 250 was found to be effective in producing fewer convergence failures due to non-normality [30].

Cronbach's alphas were computed to determine the internal consistency of the Chinese version of TEPS. Test-retest reliabilities were calculated at a 4-week interval from sample C.

## Results

### EPA and CFA

Item-total correlation analyses were performed on the 20 items of the Chinese version of TEPS in sample A of 1156 participants. Item 13, “*I don't look forward to things like eating out at restaurants. (Anticipatory)*”, had no or very low correlations with other items and was discarded in further analyses (Tables 1 and 2). Examination of the scree plot indicated a four-factor solution. Consistent with the two-factor model proposed by Gard et al [16], two of the four factors belonged to the Consummatory factor and the other two belonged to the Anticipatory factor. The two new items (19 and 20) loaded on the consummatory and anticipatory factors respectively as hypothesized.

**Table 1:** Correlation matrix of the 20 items of the Chinese translated version of TEPS in the 1156 calibration sample of Chinese participants.

	Items	a.	b.	c.	d.	e.	f.	g.	h.	i.	j.	k.
a.	TEPS1	1.00										
b.	TEPS5	0.15**	1.00									
c.	TEPS8	0.22**	0.20**	1.00								
d.	TEPS10	0.22**	0.18**	0.27**	1.00							
e.	TEPS11	0.26**	0.20**	0.30**	0.35**	1.00						
f.	TEPS2	0.12**	0.09**	0.08**	0.12**	0.13**	1.00					
g.	TEPS3	0.11**	0.19**	0.16**	0.17**	0.15**	0.52**	1.00				
h.	TEPS7	0.17**	0.15**	0.26**	0.20**	0.21**	0.28**	0.31**	1.00			
i.	TEPS9	0.13**	0.15**	0.21**	0.20**	0.22**	0.35**	0.34**	0.34**	1.00		
j.	TEPS14	0.04	0.12**	0.08**	0.13**	0.12**	0.32**	0.33**	0.27**	0.27**	1.00	
k.	TEPS19	0.13**	0.23**	0.11**	0.22**	0.14**	0.22**	0.26**	0.28**	0.23**	0.21**	1.00
l.	TEPS4	0.16**	0.17**	0.16**	0.18**	0.12**	0.33**	0.29**	0.26**	0.27**	0.19**	0.17**
m.	TEPS6	0.09**	0.14**	0.19**	0.17**	0.16**	0.30**	0.28**	0.33**	0.28**	0.14**	0.25**
n.	TEPS18	0.13**	0.12**	0.19**	0.22**	0.19**	0.22**	0.19**	0.20**	0.26**	0.23**	0.24**
o.	TEPS20	0.17**	0.11**	0.19**	0.21**	0.20**	0.17**	0.13**	0.20**	0.18**	0.14**	0.24**
p.	TEPS12	0.08**	0.17**	0.15**	0.15**	0.25**	0.11**	0.18**	0.16**	0.15**	0.13**	0.17**
q.	TEPS13	0.07*	0.05	0.02	0.02	0.04	−0.05	−0.04	−0.02	0.02	−0.15**	−0.02
r.	TEPS15	0.19**	0.17**	0.29**	0.21**	0.23**	0.22**	0.24**	0.21**	0.21**	0.21**	0.25**
s.	TEPS16	0.16**	0.15**	0.21**	0.28**	0.23**	0.21**	0.25**	0.29**	0.20**	0.18**	0.23**
t.	TEPS17	0.09**	0.21**	0.18**	0.13**	0.17**	0.21**	0.25**	0.27**	0.20**	0.30**	0.24**

	Items	l.	m.	n.	o.	p.	q.	r.	s.	t.
l.	TEPS4	1.00								
m.	TEPS6	0.40**	1.00							
n.	TEPS18	0.42**	0.42**	1.00						
o.	TEPS20	0.25**	0.24**	0.31**	1.00					
p.	TEPS12	0.11**	0.11**	0.16**	0.15**	1.00				
q.	TEPS13	−0.07*	−0.01	−0.05	−0.02	−0.08**	1.00			
r.	TEPS15	0.18**	0.19**	0.24**	0.19**	0.25**	−0.03	1.00		
s.	TEPS16	0.21**	0.17**	0.29**	0.16**	0.24**	−0.05	0.48**	1.00	
t.	TEPS17	0.13**	0.17**	0.28**	0.10**	0.23**	−0.09**	0.27**	0.26**	1.00

** *p*<0.01,

* *p*<0.05.

**Table 2:** Factor loadings of the 4-factor solution for the translated 19-item TEPS in the calibration sample.

		Factors^a^			
Items		I	II	III	IV
TEPS1	When I hear about a new movie starring my favorite actor, I can't wait to see it.(Anticipatory)		0.64		
TEPS5#	I love it when people play with my hair.(Consummatory)		0.36		
TEPS8	When I think of something tasty, like a chocolate chip cookie, I have to have one.(Anticipatory)		0.60		
TEPS10	I get so excited the night before a major holiday I can hardly sleep.(Anticipatory)		0.59		
TEPS11#	When I'm on my way to an amusement park, I can hardly wait to ride the roller coasters.(Anticipatory)		0.67		
TEPS2	I enjoy taking a deep breath of fresh air when I walk outside.(Consummatory)			0.75	
TEPS3	The smell of freshly cut grass is enjoyable to me. (Consummatory)			0.75	
TEPS7	A hot cup of coffee or tea on a cold morning is very satisfying to me.(Consummatory)			0.49	
TEPS9	I appreciate the beauty of a fresh snowfall.(Consummatory)			0.57	
TEPS14	I love the sound of rain on the windows when I'm lying in my warm bed.(Consummatory)			0.57	
TEPS19*	I love it when a baby snuggles into my arms.(Added item, to replace item 5, Consummatory)			0.28^b^	0.36
TEPS4	I look forward to a lot of things in my life.(Anticipatory)	0.67			
TEPS6	Looking forward to a pleasurable experience is in itself pleasurable.(Anticipatory)	0.68			
TEPS18	When something exciting is coming up in my life, I really look forward to it.(Anticipatory)	0.74			
TEPS20*	On the way to my first date with my beloved, I can hardly wait to see him/her. (Added item, to replace item 11, Anticipatory)	0.57			
TEPS12	I really enjoy the feeling of a good yawn.(Consummatory)				0.58
TEPS15	When I think about eating my favorite food, I can almost taste how good it is.(Anticipatory)				0.64
TEPS16	When ordering something off the menu, I imagine how good it will taste.(Anticipatory)				0.64
TEPS17	The sound of crackling wood in the fireplace is very relaxing.(Consummatory)				0.61

Notes:

a Factor I - Abstract Anticipatory; II - Contextual Anticipatory; III - Abstract Consummatory; IV - Contextual Consummatory

# indicates item part of the original TEPS;

* indicates item added to be more in line with Chinese culture.

Examination of the meaning in the items of the four factors revealed additional dimensions of the TEPS in Chinese, and the dimensions were named abstract and contextual. Therefore, the four factors of the Chinese version of TEPS were: *Abstract Anticipatory* (anticipation of something that is more abstract or broad in nature such as “I look forward to a lot of things in my life.”), *Contextual Anticipatory* (anticipation of something that is more concrete in nature such as “When I think of something tasty, like a chocolate chip cookie, I have to have one.”), *Abstract Consummatory* (consummation of emotional experience of something that is more abstract or less concrete in nature such as “I enjoy taking a deep breath of fresh air when I walk outside.”) and *Contextual Consummatory* (consummatory of emotional experience of more concrete in nature such as “When I think about eating my favorite food, I can almost taste how good it is.”). It was noteworthy that items 5 and 19 had low factor loadings on the Contextual Anticipatory factor (factor loading = 0.36) and the Abstract Consummatory factor (factor loading = 0.28), respectively. In addition, item 19 tended to have a cross factor loading on the Contextual Consummatory factors. In order to address this issue in the CFA analyses, we performed the CFA analyses with and without these two items. However, the overall fit for all studied models without these two items was not satisfactory. After consideration of the findings from both the EFA and CFA, we decided to retain these two items in the CFA analyses presented in the next paragraph.

**Figure 1:**
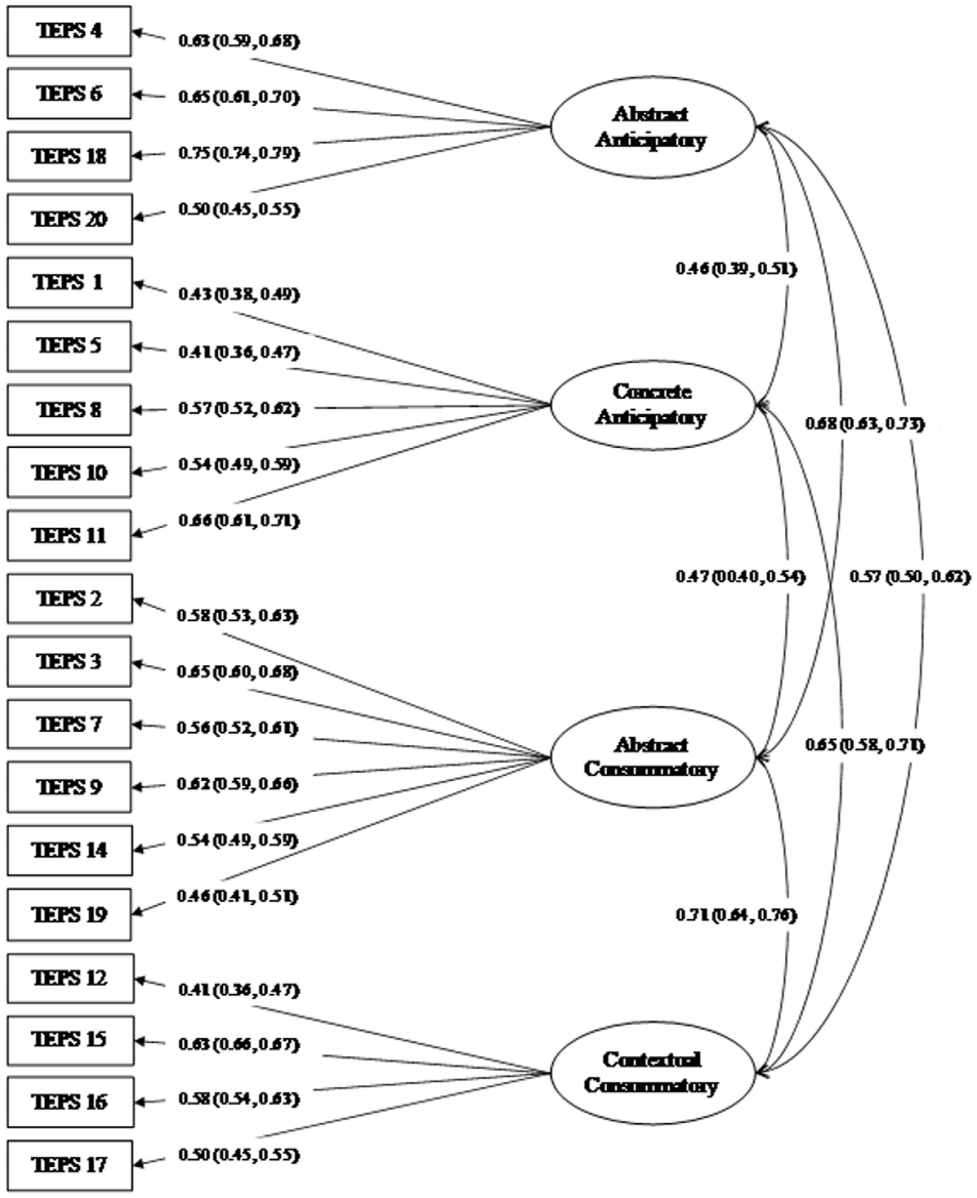
Four-factor model of the Chinese translated TEPS in the validation sample – Factor loadings and correlations between factors estimated by the Satorra-Bentler (SB) method, and their means (which is the same as the estimates by the SB method at 2 decimal places) and 95% confidence intervals (in brackets) estimated by the Bollen-Stine bootstrapping method.

In addition to the original Gard et al. [16] two-factor model and the current EFA four-factor model, a second-order four-factor model was also tested with CFA [32]. That is, the four factors of TEPS could be further factor analyzed into the original Anticipatory and Consummatory factors. All three models were tested using the SB approach with the validation sample of 1119 participants (sample B), and the four-factor model identified by the EFA yielded the best fit (Table 3). The more computationally intensive Bollen-Stine bootstrapping approach was performed only on the four-factor model. Means and 95% confidence intervals (CIs) for the fit indices and factor loadings (Figure 1 loadings plus mean+95% CI) were presented side by side with the estimates obtained by the SB approach. Estimates were within the 95% CIs unless the estimates were dependent on the sample size, which showed better results using the bootstrapping method. The two-stage factor analysis using EFA and CFA on two randomly drawn sub-samples, and the convergence of the CFA results using the SB method and the Bollen-Stine bootstrapping method provided strong empirical support for the four-factor structure of the Chinese version of TEPS.

**Table 3:** Evaluative measures of model fit by the three models, and the Bollen-Stine bootstrap estimates for the 4-factor model in the 1119 validation sample of Chinese participants.

	Confirmatory factor analysis			BS bootstrapping 4-factor model^c^	
Good-of-fit indices^a^	2-factor model^b^	4-factor model	2nd-order factor model	Mean	95% CI^d^
SB scaled χ^2^	1065.276	452.2996	482.7125	176.2968^e^	(142.94, 219.95)
df	151	146	147	146	-
*p*	<0.001	<0.001	<0.001	0.1466^f^	(0.0001, 0.5562)
GFI	0.8916	0.9512	0.9482	0.9838	(0.9799, 0.9868)
AGFI	0.8636	0.9366	0.933	0.9789	(0.9739, 0.9828)
SRMR	0.06124	0.04184	0.04511	0.0228	(0.0201, 0.0257)
NFI	0.897	0.9563	0.9533	0.9583	(0.9475, 0.9672)
NNFI	0.8982	0.9647	0.9616	0.9912	(0.9790, 1.0008)
CFI	0.9101	0.9699	0.967	0.9924	(0.9821, 1.0000)
RMSEA	0.07359	0.04332	0.0452	0.0124	(0.0000, 0.0213)
*p* (RMSEA<0.05)	<0.001	0.9924	0.9602	-	-

Notes:

a SB scaled χ^2^ - Santorra-Bentler scaled χ^2^; df - degree of freedom; *p* - *p* value; GFI - Goodness-of-fit index; AGFI - Adjusted GFI; SRMR - Standard Root Mean Square Residuals; NFI - Normed Fit Index; NNFI - Non-normed Fit Index; CFI - Comparative Fit Index; RMSEA - Root Mean Square Error of Approximation.

b Kring et al. (2004).

c Based on 250 bootstrap sample of size 250.

d 95% Confidence Interval.

e Model χ^2^ instead of SB scaled χ^2^.

f 127 of 250 bootstrap samples with p>0.05.

### Internal consistency and test-retest reliability

Samples A and B were merged to calculate the internal consistency. Cronbach's α of the four factors were as follows: Abstract Anticipatory = 0.69; Contextual Anticipatory = 0.63; Abstract Consummatory = 0.72; Contextual Consummatory = 0.60. Internal consistency for the total scale was 0.83. Test-retest reliabilities were very good (r = 0.79 to 0.81, p<0.0005) from sample C, suggesting that the measure is assessing stable trait-like experiences.

## Discussion

Through testing two alternative models: the original two-factor model by Gard et al. [16] and a four-factor model, we found that the four-factor model, namely the anticipatory contextual, anticipatory abstract, consummatory contextual, and consummatory abstract, was a better fit to the current study's large Chinese sample. The current findings were broadly consistent with Western findings that subjective experience of pleasure can be classified into consummatory and anticipatory components [16], [17]. However, our findings further showed that both the consummatory and anticipatory factors could be further sub-divided into abstract and contextual factors in the Chinese context.

Because selecting information from memory to represent the future is highly relevant to an individual's goals, beliefs and concerns [33], it is possible that frequency of events will facilitate access to reaching them in memory. For the consummatory aspect of the TEPS, those contextual items refer to more physical feelings such as satiation to food, may occur more frequently during daily life than more abstract items. If these experiences are more readily recalled, they may be perceived as subjectively closer to the present by Chinese individuals. The focus on food and eating, an obviously common experience, may also help explain why two anticipatory items from the original TEPS fit on the Contextual Consummatory factor in the Chinese context.

Anecdotally, Chinese respondents took longer to rate the anticipatory abstract items of the TEPS which described very general events. Perhaps it was more difficult for them to envision a non-specific event since they relied on past experience to answer contextual items and were less able to do so for abstract items. However, since the previous studies in other countries did not take the account the response time to rate the anticipatory items, we do not know whether it is really more difficult for Chinese participants to envision a non-specific event than their Western counterparts.

It is important to note that the current findings are not directly comparable to the Western studies of the TEPS because there are differences in statistical approach and other methodological factors (e.g., nature of the study samples). Indeed, given the complexity of cultural differences, and the absence of any non-Chinese comparison group, the current study only provides preliminary evidence for cross-cultural aspects to the temporal experience of pleasure. Without comparable cross-cultural samples between Chinese and Westerner counterparts, we are not confident that the 4 factors generated from the current Chinese sample reflect true cultural differences. The current findings might be either due to cultural or language factors (along with sampling bias and measurement error). Further studies using more rigorous approaches and objective methods such as behavioral experiments [17] and brain imaging paradigms [34] are required to further validate these potential cross-cultural variations of the temporal experience of pleasure. Similarly, it is noteworthy that the labels of “abstract” versus “contextual” appear appropriate for the anticipatory items. However, this dichotomy does not seem to fit as well in distinguishing the two consummatory factors. That is, the items on the “abstract” consummatory factor may also refer to specific contextual pleasures. Indeed, the idea of a consummatory pleasure may be inherently contextual. Moreover, future studies should expand the healthy group size and include community samples in order to cross validate the current factors, particularly to distinguish the two consummatory factors, and to determine whether this Chinese version of TEPS, which includes one more directly social anticipatory item than the original TEPS, will maintain its usefulness in community settings.

In a preliminary study of the TEPS in Chinese patients with schizophrenia [22], we found that patients with minimal negative symptoms experienced significantly more pleasure in contextual anticipatory and contextual consummatory factors than negative symptom patients. Those patients with minimal negative symptoms also exhibited a tendency to experience more abstract anticipatory pleasure than negative symptom ones but there was no significant difference in abstract consummatory pleasure. TEPS scores were also inversely associated with clinical symptom levels [22]. However, since the patients we studied were a relatively chronic sample of patients, we do not know whether a similar deficit would be demonstrated in patients at the early stage of the illness. Therefore, studies are needed that include a wider range of patients at different stages of the illness and different clinical diagnoses (i.e., major depressive and bipolar disorders) with specific anhedonia symptoms in order to determine clinical discrimination across clinical diagnoses.

Taken together, the current study is the first one to examine the factor structure of the TEPS in a Chinese sample. The TEPS is specifically developed to capture two faces of trait hedonic capacity and has been demonstrated with impressive latent structure for researchers to study hedonic trait in healthy Chinese participants.
